# Bioanalysis in the Age of New Drug Modalities

**DOI:** 10.1208/s12248-021-00594-w

**Published:** 2021-05-03

**Authors:** Jing Shi, Xuesong Chen, Jianbo Diao, Liying Jiang, Lan Li, Stephen Li, Wenzhong Liang, Xiaoying Jin, Yonghui Wang, Colton Wong, Xiaolong Tom Zhang, Francis L.S. Tse

**Affiliations:** Bioanalytical Services Department, WuXi AppTec, 288 Fute Zhong Road, Waigaoqiao Shanghai, 200131 China

**Keywords:** New drug modality, bioanalysis, immunogenicity, oligonuleotide, cell and gene therapy

## Abstract

In the absence of regulatory guidelines for the bioanalysis of new drug modalities, many of which contain multiple functional domains, bioanalytical strategies have been carefully designed to characterize the intact drug and each functional domain in terms of quantity, functionality, biotransformation, and immunogenicity. The present review focuses on the bioanalytical challenges and considerations for RNA-based drugs, bispecific antibodies and multi-domain protein therapeutics, prodrugs, gene and cell therapies, and fusion proteins. Methods ranging from the conventional ligand binding assays and liquid chromatography-mass spectrometry assays to quantitative polymerase chain reaction or flow cytometry often used for oligonucleotides and cell and gene therapies are discussed. Best practices for method selection and validation are proposed as well as a future perspective to address the bioanalytical needs of complex modalities.

## INTRODUCTION

With the advances in genomics and a deeper understanding of the biological pathways linked to human diseases, the paradigm of discovery and development of novel pharmaceutical therapies is quickly shifting from intervention of conventional protein targets to those previously considered “undruggable” such as specific genes, deoxyribonucleic acid (DNA)/ribonucleic acid (RNA), and protein-protein interactions (1). Novel target identification technologies, combined with emerging new drug modalities beyond classic small molecules, monoclonal antibodies, and antibody drug conjugates, have expanded the repertoire of options to unlock new solutions for unmet medical needs (2, 3).

As with conventional drugs, the assessment of safety and efficacy of the new therapeutic modalities requires a thorough understanding of their pharmacokinetic (PK) and toxicokinetic (TK) characteristics. New bioanalytical strategies and platforms are needed not only to measure the parent drug and relevant metabolites (4, 5) but also to evaluate potential immunogenicity which is often associated with the novel therapies. Whereas traditional bioanalytical methods typically rely on liquid chromatography-mass spectrometry (LC-MS) and ligand binding assays (LBA) for small molecules and biologics, respectively (6), hybrid LBA/LC-MS and multiple LBA/LC-MS approaches have been applied to measuring complex large molecules (7, 8). Among the new drug modalities, RNA, cell, and gene therapies are non-protein drugs in nature; thus, technology platforms other than LC-MS and LBA, such as quantitative polymerase chain reaction (qPCR), sequencing, hybrid LBA, or flow cytometry, are necessary to measure the molecular or cellular drug form.

Whereas regulatory guidelines for the new drug modalities are yet to be developed, bioanalytical strategies have been implemented based on understanding of the drug’s molecular structure, functionality, biotransformation, and immunogenicity. The present review highlights the various bioanalytical challenges and considerations for six types of new modalities: RNA-based drugs, bispecific antibodies and multi-domain protein therapeutics, prodrugs, gene therapies, cell therapies, and fusion proteins.

## RNA-BASED DRUGS

Oligonucleotides are single-stranded DNA/RNA or DNA/RNA analogs that can be classified based on their mechanism of action into antisense oligonucleotides (ASOs), small interfering RNAs (siRNAs), microRNAs (miRNAs), and aptamers (9). In addition, numerous decoys, synthetic guide RNAs (sgRNAs), mRNAs, and immunostimulatory oligonucleotides also belong to this category (10). As of December 2020, twelve oligonucleotide-based drugs have been approved by the US FDA (Table I). Because of the diverse nature of these RNA-based drugs, no single bioanalytical approach can fulfill the requirements for quantification of this drug class. Multiple analytical techniques including qPCR, hybridized immunoassay, and LC-MS-based methods have been used, each with its unique advantages and shortcomings (Table II) (11).

**Table 1:** Therapeutic Oligonucleotides Approved by the US FDA as of December, 2020

Approval year	Generic name	Trade name	Class	Sponsor	Dose route	Indication
1998	Fomivirsen	Vitravene	21mer ASO	Ionis/Novartis	Intravitreal	Cytomegalovirus (CMV) retinitis
2004	Pegaptanib	Macugen	Aptamer	NeXstar	Intravitreal	Age-related macular degeneration
2013	Mipomersen	Kynamro	20mer ASO	Ionis/Genzyme	Subcutaneous	Homozygous familial hypercholesterolemia
2016	Defibrotide	Defitelio	Mixture of DNA strands	Jazz	Intravenous	Hepatic veno-occlusive disease
2016	Eteplirsen	Exondys 51	30mer ASO	Sarepta	Intravenous	Duchenne muscular dystrophy (DMD)
2016	Nusinersen	Spinraza	18mer ASO	Ionis/Biogen	Intrathecal	Spinal muscular atrophy
2018	Patisiran	Onpattro	siRNA	Alnylam	Intravenous	Polyneuropathy caused by hereditary transthyretin amyloidosis
2018	Inotersen	Tegsedi	20mer ASO	Ionis/Akcea	Subcutaneous	Polyneuropathy caused by hereditary transthyretin amyloidosis
2019	Givosiran	Givlaari	siRNA	Alnylam	Subcutaneous	Acute hepatic porphyria
2019	Golodirsen	Vyondys 53	25mer ASO	Sarepta	Intravenous	Duchenne muscular dystrophy (DMD)
2020	Viltolarsen	Viltepso	ASO	Nippon Shinyaku	Intravenous	Duchenne muscular dystrophy (DMD)
2020	Lumasiran	Oxlumo	siRNA	Alnylam	Intravenous	Primary hyperoxaluria type 1 (PH1)

*ASO *antisense oligonucleotide*, DNA *deoxyribonucleic acid*, siRNA *small interfering ribonucleic acid

**Table 2:** Comparison of Quantitative PCR (qPCR), Hybridization Immunoassay, and MS-Based Methods for RNA/DNA Quantification

Technique	qPCR	Hybridization immunoassay	MS
Instrumentation	Real-time PCR	Plate reader; MSD for hybridized MSD	LC-MS
Sample pretreatment	Nucleic acid extraction needed	Method specific	Extraction needed
Sensitivity	Highest	High	Acceptable; study specific
Specificity	Endogenous interference,potential contamination	Endogenous interference	Highly specific
Reagent	Probe customizationand common reagents	Probe customization	Common reagents and consumables
Regulatory guidance onmethod validation	Not available	Ligand-binding assays	LC-MS

*PCR* polymerase chain reaction, *LC-MS* liquid chromatography-mass spectrometry, *RNA* ribonucleic acid, *DNA* deoxyribonucleic acid, *MSD* Meso Scale Discovery

### Bioanalysis Platforms and Considerations

#### qPCR Assay

PCR technologies including reverse transcription PCR (RT-PCR) and qPCR are modern molecular technologies applied to amplify and detect DNA and RNA sequences using oligonucleotide primers, deoxyribonucleoside triphosphates (dNTPs), and heat-stable DNA/RNA polymerase. qPCR is highly sensitive and precise and covers a broad dynamic range. However, when the DNA/RNA fragment is shorter than 18bp, specific hybridization binding will be inefficient, and off-target interaction will be high, rendering qPCR an inappropriate methodology (12). To overcome the difficulty in amplifying short RNA fragments of siRNA or miRNA, numerous approaches including primer extension (PE), invader assay, stem-loop RT-PCR, ligation assay, and competitive qPCR have been developed (9). The PE assay utilizes a gene-specific primer to reverse transcribe RNA into cDNA, followed by qPCR amplification with a reverse primer containing locked nucleic acids (13). Stem-loop RT-qPCR adopts a custom-designed stem-loop primer which hybridizes with siRNA/miRNA and reverse transcribes it into cDNA using transcriptase, after which standard Tagman qPCR is applied to quantify the RT products (14). The competitive qPCR method is based on the competition between siRNA and homologous DNA for binding to template DNA (13). Unlike in standard qPCR, DNA primers are not present in excess; thus, the competitive binding of siRNA to the template could be calculated. The use of qPCR on oligonucleotides has been extended to aptamers that have a relatively long sequence (15). Chemical modification of an ASO, siRNA, and aptamer could improve the drug’s stability and uptake efficiency but may potentially interfere with qPCR primer annealing, amplification efficiency, and accuracy and precision of the method (12).

#### Hybridization Immunoassay

In contrast to qPCR, hybridized immunoassays provide comparable sensitivity with significantly enhanced throughput. A typical design of the hybridization oligonucleotide sandwich consists of the capture and the detection probes. The analyte in a matrix is initially denatured and hybridized with the capture probe. The hybridized complex is then attached to a plate through biotin association. Subsequently, the detection probe is mixed, incubated, and washed to remove any non-ligated fraction. The truncated duplex is cleaved, substrate is added, and signal is read on a fluorescence plate reader (16–19). The development of locked nucleic acid (LNA) probes for hybridization provides enhanced specificity to overcome endogenous interference or cross-reactivity that conventional immunoassays often encounter. Detection sensitivity and dynamic range can be bolstered further by moving from the standard fluorescent reader to the Meso Scale Discovery (MSD) electro-chemiluminescent platform (20).

#### MS-Based Methods

The advantages of LC-MS-based techniques include high specificity, excellent accuracy and precision with high reproducibility, and wide dynamic ranges. For the quantification of oligonucleotides, a variety of sample preparation approaches such as Trizol extraction (liquid-liquid extraction), proteinase K digestion, solid-phase extraction, immunocapture, or hybridization of these pretreatment strategies have been well studied (21). With the growing popularity of high-resolution mass spectrometry (HRMS), the LC-high-resolution accurate mass (LC-HRAM) assays bring about simultaneous metabolite identification and quantification of oligonucleotide. Superior specificity can be achieved by adjusting the resolution of the mass spectrometer to differentiate endogenous interference and potential metabolite(s) in the biological matrix. When full scan detection mode is employed, without having extensive prior knowledge of the oligonucleotide, parent drug quantification and metabolite exploration both can be achieved via mining of the acquired data without multiple experimentation (22). On the downside, the sensitivity of LC-MS-based methods has never matched those achieved by qPCR or hybridization immunoassay. Furthermore, the development of the LC program, usually specific to each oligonucleotide, requires a certain experience on the selection of ion-pairing reagent combination. On the other hand, the use of hybridization-based LC-fluorescence assays could achieve detection limits comparable to hybridized immunoassays while maintaining the simplicity of sample preparation (23).

### Immunogenicity Considerations

Immunogenicity is a critical factor in the clinical development of biological therapeutics. The biologic may induce an immunologic response and the generation of anti-drug antibodies which will affect the pharmacologic and/or toxicologic effect(s) of the product. Compared to protein therapeutic products, the immunogenicity of oligonucleotides is relatively low due to their biological and chemical nature (24). Oligonucleotides are structurally related to nucleic acids (DNA and RNA) that tend to be low in immunogenicity (25), and they are smaller in size and have fewer immunogenicity epitopes than their protein analogs. Nonetheless, oligonucleotides can trigger an immune response by interacting with internal DNA sensors such as toll-like receptors; thus, the possible production of anti-drug antibodies needs to be examined. Currently, there is no consensus on the strategy and critical parameters for an oligonucleotide immunogenicity evaluation assay, although some general recommendations have been proposed (26).

## BISPECIFIC ANTIBODIES (BsAb) AND MULTI-DOMAIN PROTEIN THERAPEUTICS

**Fig. 1:**
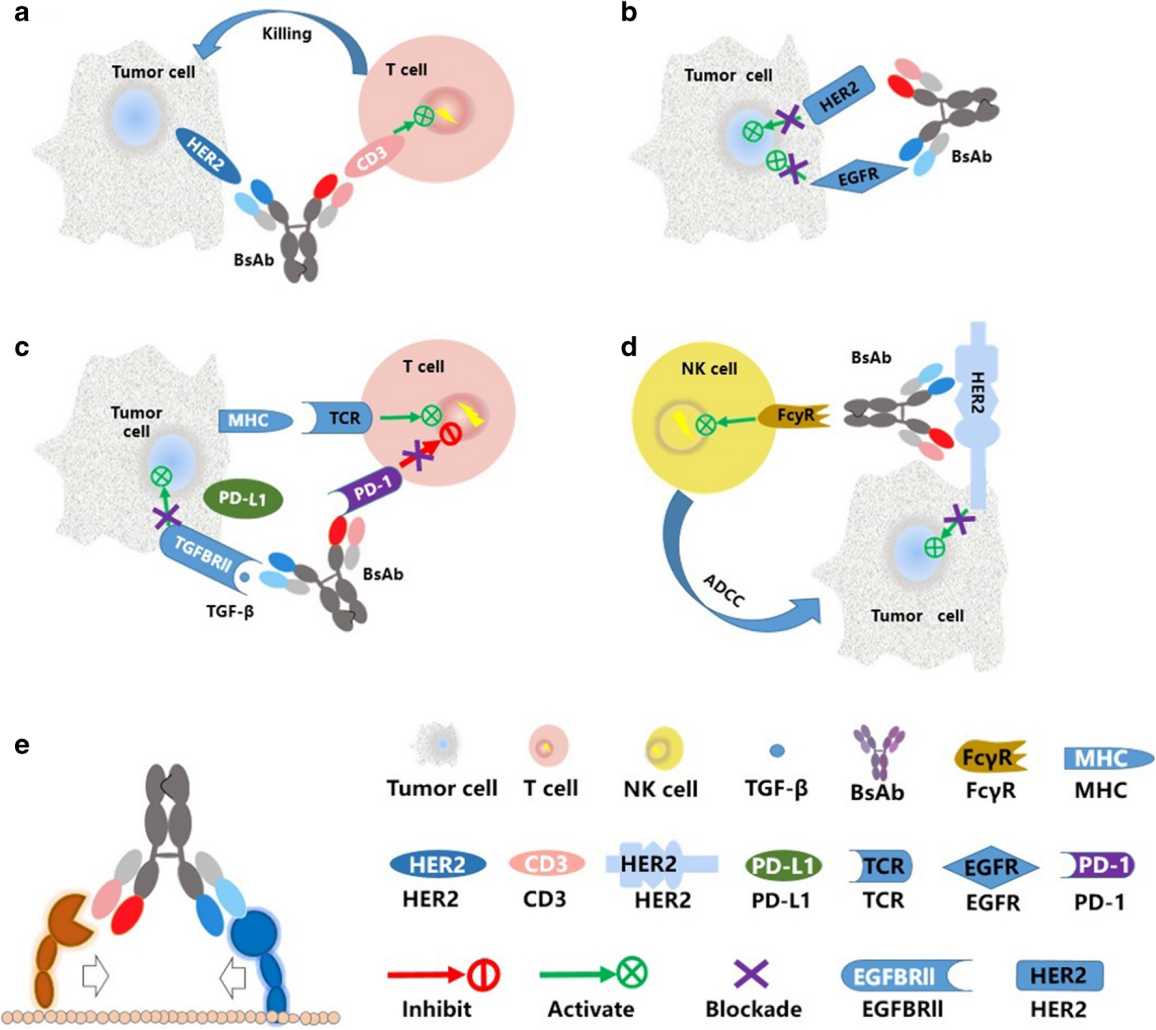
Mechanisms of action of bispecific antibodies according to their structures and targets. **a** Bridge cells and immunomodulation by targeting cancer cells and immune cells simultaneously to kill cancer cells by the cytotoxicity of immune cells. **b** Dual signaling inhibition targets two different receptors with the blockade of two signal pathways to suppress tumor activity. **c** Co-localized blocking via inhibition of the tumor activity by both tumor cell-intrinsic and cell-extrinsic pathways. **d** Biparatopic bispecific antibodies target two different epitopes of the same antigen to enhance the affinity of BsAb and target. **e** Formation of protein complexes by precision direction of enzyme and substrate as a cofactor mimetic

Bispecific antibodies are recombinant antibodies generated by chemical crosslinking, hybridoma technology, or genetic engineering that consist of two distinct binding domains capable of recognizing two different antigens or two different epitopes of the same antigen (27). Whereas more than 85 formats of BsAb have been reported, they can be classified into two categories: those bearing an Fc region and those lacking an Fc region (28, 29). The Fc-bearing, IgG-like forms, with Fc-mediated effector functions such as antibody-dependent cell-mediated cytotoxicity (ADCC), complement-dependent cytotoxicity (CDC), and FcRn-mediated recycling, tend to enhance drug efficacy. Various technologies have been used for the design of BsAbs (30–39). The mechanisms of action of BsAbs such as targeting cancer cells and immune cells simultaneously to facilitate elimination of cancer cells, blockade of two signal pathways to suppress tumor activity, etc. can be summarized into five categories as shown in Fig. 1 (40–44). There are more than 110 BsAbs in active clinical development (45, 46).

### Bioanalytical Considerations

The complex structures and mechanisms of action of BsAbs have led to bioanalytical challenges, and a mixture of the active and inactive forms of the BsAb in the body may need additional considerations. The FDA guidance for BsAb development (47) states that multiple PK assays may be needed to quantify the level of total, bound, and unbound BsAb, and multiple immunogenicity assays may be needed to measure immune responses to different domains of a BsAb. At the minimum, an assay detecting the intact form of the BsAb, which is the active form with the two unbound binding sites, is necessary. This can be achieved by designing a single assay recognizing the two functional domains of the BsAb by the two target antigens or antibodies. If the affinity between the functional domains and antigens is too low to provide acceptable assay sensitivity or if target interference is a concern, anti-idiotype antibodies would be preferred.

Due to the bispecific nature of BsAbs, the PK assays can be designed in three different formats with specific reagent pairs (capture and detection): 1) intact assay with one target/anti-idiotype antibody for one domain as capture and the other as detection, 2) total form with a pair of antibodies against the framework or scaffold, 3) mono-functional assay with a pair of target/anti-idiotype and anti-human IgG (48). Some unique considerations during assay design for BsAbs include 1) stability of intact form in the matrix. Mono-functional assay often can provide additional information on drug stability; reagents including dithiothreitol (DTT) and cocktail of enzyme inhibitors or conducting sample pretreatment on ice may resolve the issue. 2) High sensitivity requirement. Due to the high potency and, therefore, low clinical doses of BsAbs, a sensitive PK assay with LLOQ at pg/mL level is often needed in clinical studies. 3) Steric effect in the selection of reagents for Fc-lack BsAbs.

### Immunogenicity Considerations

**Fig. 2:**
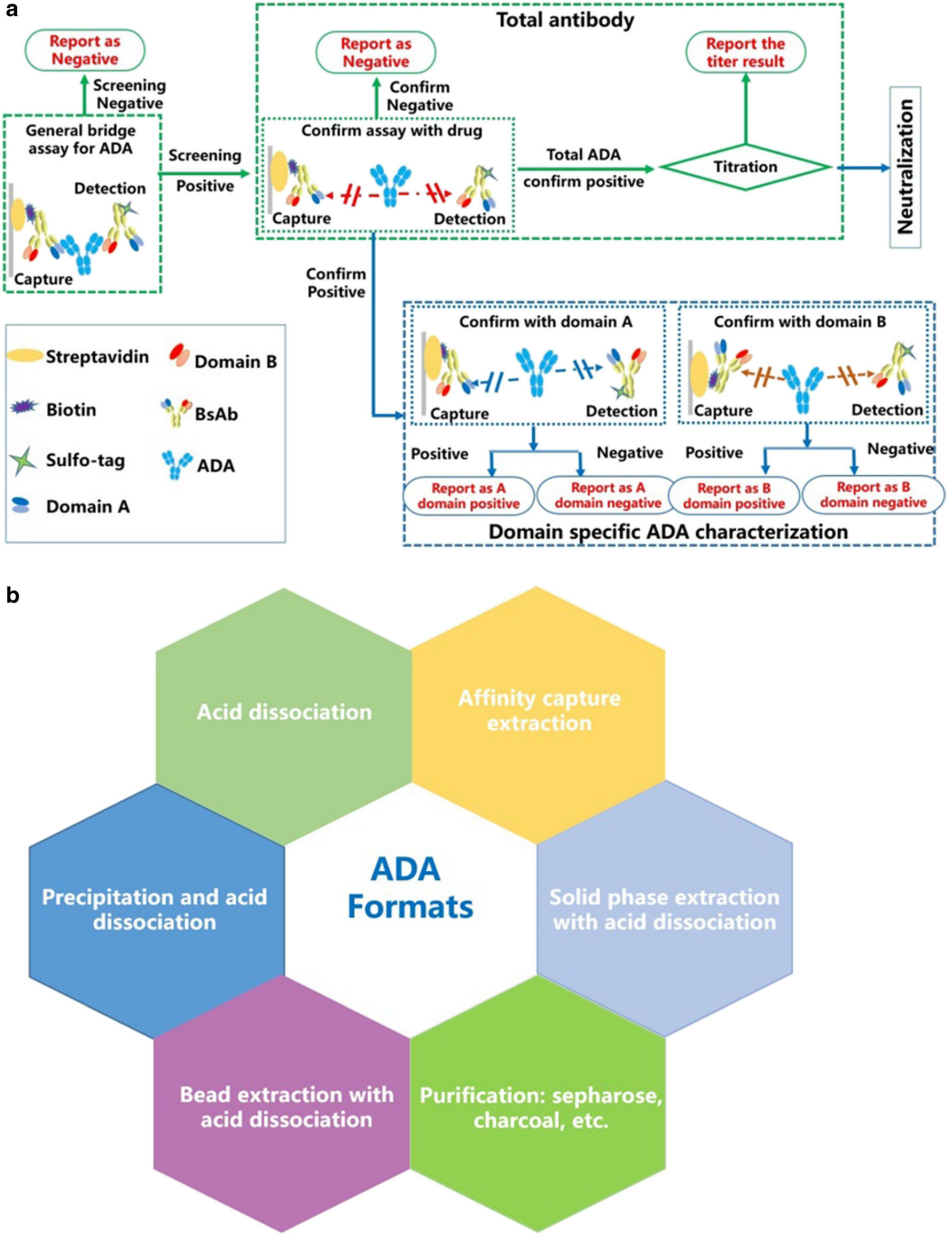
Strategy of immunogenicity for bispecific or multi-functional biotherapeutics. **a** Flow chart of ADA analysis for screening, confirmatory, and titration (green dotted-line box) and characterization for domain-specificity (blue dotted-line box) with the corresponding competing compounds in the confirmatory assay. **b** Different formats of immunogenicity assay including acid dissociation, ACE (affinity capture extraction), SPEAD (solid phase extraction with acid dissociation), and precipitation with polyethylene glycol (PEG) and other purification solutions using sepharose or charcoal

Since anti-drug antibody (ADA) is mainly induced by exogenous components or sequence, immunogenicity is a particularly important concern for BsAbs which bear more exogenous or unnatural sequences (46). Characterization of domain specificity based on the structures, epitopes, and flexible linkers is also important to assess the safety and efficacy of BsAbs. An analytical stream for ADA evaluation from screening to characterization as recommended by the FDA (49) is shown in Fig. 2a. The need for domain-specific ADA and neutralizing (NAb) assay may vary at different stages of drug development. For example, for the interpretation of toxicity data in nonclinical studies, it would be sufficient to run the ADA assay following the traditional 3 tiers for total ADA. In clinical studies where immunogenicity results are used to assess the impact on PK/PD and treatment efficacy as well as adverse events, the identification of domains and neutralization assessment are critical, and at least one NAb assay indicating the primary therapeutic mechanism of action (MoA) is needed (50). Most BsAbs achieve their efficacy by the synergistic effect of binding to two cell surface targets simultaneously; thus, a cell-based NAb assay sensitive to neutralizing binding to either target is adequate. This can be a functional assay measured by a plate reader or flow cytometer. Alternatively, genetically engineered cell lines with a reporter gene as an endpoint to be measured have been employed for this purpose.

Given the complicated and variable making and structure of BsAbs, the conventional bridge assay may be unable to deliver both sensitivity and tolerance, in which case a diversity of assay formats (Fig. 2B) should be explored. Large differences in signal readout were observed between naïve individuals during the screening phase of method development for IgG-scFv-based compounds, and trying multiple assay formats and buffers yielded little improvement until a higher MRD (minimum required dilution) and target interference exclusion by immune capture beads were utilized. Others have reported the same phenomenon (51). For those BsAbs with the two functional binding sites distributed in tandem on the CDR (complementarity determining region) of each arm, the two arms of a given ADA molecule would bind to the same BsAb molecule, thus failing to form a bridge complex by simultaneous binding to two drug molecules. Instead of a bridge assay, one could use an alternative format in which the drug is pre-coated on a microplate to capture ADAs and anti-human IgG/IgM is used as detection.

## PRODRUG

For oncology programs, antibody-based biotherapeutics are designed to target antigens highly expressed on specific tumor cells. Often, these antigens are also expressed on normal cells which can incite systemic autoimmunity such as Cytokine Release Syndrome (CRS), resulting in life-threatening “on-target, off-tumor” toxicities (52). One solution to overcome this limitation is to use the prodrug form of the biotherapies (53, 54). Upon administration, the prodrug remains inactive in the systemic circulation but becomes active after reaching the tumor microenvironment (TME). The TME has a few features that differ from most normal tissues, including a lower extracellular pH environment (55, 56), overexpressed proteases (57), and tumor-specific cell-surface proteins. A typical antibody prodrug contains a protease-cleavable peptide linker between the functional therapeutic body and the masking blocker so that activation can occur in the presence of overexpressed proteases such as matrix metalloproteases (MMPs) in TME. This strategy has been employed to engineer biotherapeutic prodrugs for many cancers and other diseases, and five major formats of prodrugs are currently undergoing research and development (58), namely 1) naked monoclonal antibody (mAb) based, 2) antibody-drug-complex (ADC) based, i.e., pro-antibody drug conjugates (PDC), 3) T cell-engaging bispecific antibodies (TCB) based, 4) chimeric antigen receptor T cell (CAR-T) based, and 5) cytokine based.

The prodrug design complicates the structures of the biotherapeutics, increases the number of metabolic variants, and implies a low concentration of cleaved forms in the systemic circulation with a large number of similar antibodies present, all of which pose huge challenges to the bioanalysis of this new drug modality. Although the MoA of prodrug is to minimize the generation of activated form of prodrug and formation of target-bound drug in the systemic circulation, some circulating, cleaved form of drug will be expected after repeated dosing due to either activation by protease in circulation or escape from TME after site activation. This may not be an issue in preclinical studies using non-tumor-bearing animals but can be problematic in clinical studies in diseased patients. The potential need to distinguish the target-bound from unbound forms should be considered. Extra care should be taken in the case of T-cell-engaging bispecific (TCB) antibody because of its two different target-binding domains. As shown in Table III, multiple bioanalytical platforms are needed to address different biological questions raised during the life cycle of drug research and development.

**Table 3:**
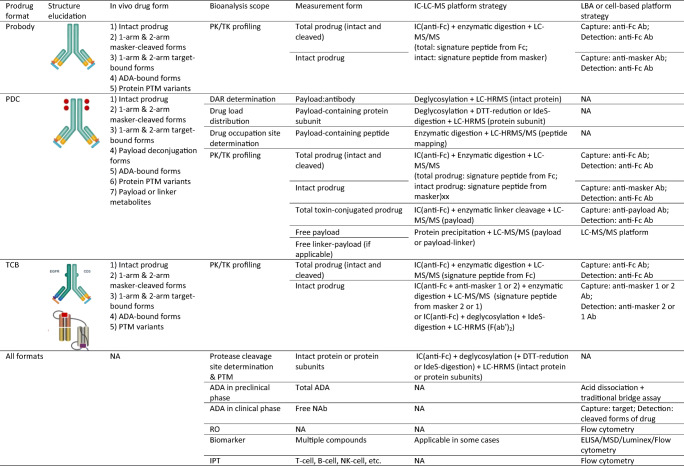
Bioanalysis Platforms for Antibody Prodrugs

*PDC* pro-antibody drug conjugates, *TCB* T cell-engaging bispecific antibodies, *IC-LC-MS* immunocapture-liquid chromatography coupled with tandem mass spectrometry, *LBA* ligand binding assay, *ADA* anti-drug antibody, *PK/TK* pharmacokinetic/toxicokinetic, *HRMS* high-resolution mass spectrometry, *DTT* dithiothreitol, *MSD* Meso Scale Discovery, *PTM* posttranslational modification, *DAR* drug antibody ratio, *RO* receptor occupancy, *IPT* immunophenotyping, *ELISA* enzyme-linked immunosorbent assay

### Bioanalysis Platforms and Considerations

#### Ligand Binding Assay (LBA)

Although LBA offers great sensitivity and high throughput, in the case of antibody prodrugs, various specific antibody reagents targeting the different functional domains are needed which will significantly increase turnaround time (TAT) and cost, and multiple assays are required to measure various drug forms *in vivo* because of the monoplex nature of LBA. Taking mAb of prodrug as an example, in order to detect the intact form, a specific anti-masker antibody can be used for coating and an anti-Fc antibody can be used as detection antibody. The specificity of this anti-masker antibody is essential to avoid cross reaction with the cleaved form. Interference also can be caused by possible dynamic binding between free masker and cleaved form in the samples. On the other hand, in order to detect the cleaved form, a capture antibody that is specific to the cleaved form, probably CDR (complementarity determining region) of the cleaved form is needed, and an anti-Fc antibody can be used as detection antibody. Alternatively, instead of measuring the cleaved form, an approach of measuring total antibody drug concentration can be used where anti-Fc antibody can be used as both capture and detection antibodies. Actually, in PK or TK profiling, “intact and total” approach is preferred to “intact and cleaved” approach because it is rational to expect a very low concentration of cleaved forms in the systemic circulation due to the designed feature of TME activation of a prodrug.

#### Immunocapture-Liquid Chromatography Coupled with Tandem Mass Spectrometry (IC-LC-MS/MS) or HRMS

IC-LC-MS/MS has an exceptional advantage when applied to antibody prodrug due to its great selectivity, wide dynamic range, multiplexibility, and less limitation by the availability and specificity of reagents. Under most circumstances, only a generic capture antibody such as anti-Fc or protein A/G is needed in IC, and selectivity is greatly improved by downstream LC separation and MS filtration, either on peptide level (LC-MS/MS) or protein level (HRMS). The multiplex feature of LC-MS also greatly reduces the number of assays needed. However, the capture efficiency needs to be optimized when using a generic capture antibody which usually pulls down both prodrug and IgG from animal species, especially monkey in preclinical studies and endogenous IgG of patients in clinical studies.

HRMS can measure intact protein with functional information and distinguish small mass shifts of biotransformational variants via its extremely high mass resolving power, hence serving as an important tool for protein characterization (59). In comparison with LBA, a common drawback of LC-MS assay is its lower sensitivity due to limited choices of signature peptide of the intact form. Improvements are possible by trying different digestive enzymes, alkylation reagents, peptide derivatization or HILIC/microLC/nanoLC setup, or a method called Stable Isotope Standards and Capture by Anti-Peptide Antibodies (SISCAPA) (60). In LC-HRMS assay, reducing the protein size into protein fragments by disulfide reduction or FabRICATOR® (IdeS) digestion can help to improve the sensitivity.

The automation of sample preparation is important in an IC-LC-MS/MS platform to achieve high reproducibility and minimize human errors. Currently available automation platforms (KingFisher ^TM^ Flex 96, Agilent AssayMAP, ThermoFisher’s Versette ^TM^, and Tecan Freedom EVO®) solely focus on immunocapture steps, whereas the integration of immunocapture with downstream enzymatic digestion steps is highly desirable and may ultimately improve sample throughput and method quality.

There are no regulatory guidelines for method validation of large molecular bioanalysis using LC-MS platform, but a few White Papers have been published on this topic (61–63). At present, the general opinion is that the same acceptance criteria used in LBA can be applied for hybrid LC-MS assay. In addition to the conventional tests in method validation for chromatographic assays, the stability and lot-to-lot variance of critical reagents, capture efficiency, enzymatic digestion efficiency, and interference between the measured subunits of intact protein must be determined in an IC-LC-MS/MS assay.

#### Capillary Electrophoresis (CE) Immunoassay

CE immunoassay is a variant of LBA where the ligand binding mechanism is used for protein drug purification and detection, with upstream CE separation based on either protein size or protein charge (64). It is similar to LBA in sensitivity even with nano-liter sample volume but lower in throughput (~19 h for 96 samples). The size resolving power of CE is limited, the data in charge-separation mode are sometimes complicated and difficult to interpret, and the instrument, the Simple Western system (by Protein Simple), is relatively expensive though fully automated. The method may also be applied to protein biomarkers and metabolite identification as well as anti-drug antibody (ADA) analysis.

### Immunogenicity Considerations

Prodrugs, especially the TCB version as modified non-native bispecific molecules, are prone to immune response and can induce a large amount of ADA *in vivo*. The cleavage of the masking peptide of prodrug may generate new epitopes. The immunogenicity considerations for the prodrug of TCB are similar in principle to those for BsAb but more complicated because each ADA assay of BsAb now yields two versions, one for intact prodrug and another for the cleaved form. As a result, two total ADA assays (for intact and cleaved forms) are recommended to facilitate understanding of the immunogenicity potential of the exposed target-binding sites on the cleaved format. For the NAb experiment, in addition to the conventional NAb assay for BsAb, the ADA that blocks the binding site of the linker and activating protease also should be considered as it could inhibit the activation of the prodrug and thus impair drug efficacy.

## GENE THERAPY (GT)

Gene therapeutics are a special class of drugs that are composed of vectors and transgenes. The vector delivers the transgene to the nucleus of target cells where the target protein is expressed for therapeutic purposes. During the development of GT products, biodistribution, shedding, and immunogenicity are three critical parameters in assessing drug safety and efficacy across all development phases. This section reviews the current practices, challenges, and considerations in designing the bioassays for supporting the development of GTs.

### Bioanalytical Evaluation of Biodistribution

Biodistribution studies are needed to assess the distribution, persistence, and clearance of the vector and possibly the expressed transgene product, from the site of administration to target and non-target tissues and biofluids (65). These data can determine the extent of tissue transduction and transgene expression, evaluate whether expression is transient or persistent, and guide the design of preclinical toxicology studies as well as early phase clinical trials. The characterization of viral vector and transgene is often supported using two different qPCR tests. Developing a PCR assay involves the extraction of nucleic acid followed by the amplification and detection of target nucleic acids. Despite the prevalence of qPCR, this approach has a few disadvantages. It requires a large number of target cells to extract sufficient mRNA for quantifying the gene expression. Additionally, qPCR is limited in its ability to quantify gene expression using heterogeneous cell preparations and to concurrently measure mRNA and protein (66). Flow cytometry for the measurement of mRNA and protein is an alternative in characterizing the biodistribution of GTs (67), though its sensitivity is not comparable to that of qPCR. A few other platforms such as LC-MS and ligand binding assay (LBA) have been developed for detecting the translational protein. In the LC-MS approach, the analyte of interest is extracted from the study samples through various protein precipitation protocols prior to analysis using LC-MS. The selection of protein precipitation protocol depends on multiple considerations including the properties of target protein and the availability of target protein binding reagents, the type of biomatrix, the analyte extraction protocol, and the status of measured analyte (i.e., free or total form) and the performance of key assay parameters (i.e., sensitivity and specificity). LC-MS has a long history in supporting the protein quantification with many advantages such as fast method development, high specificity, and multiplexing capability to measure metabolites in various biomatrices (68). However, the LC-MS approach may not be able to support the GTs with low-level translational protein expression due to limited sensitivity. LBA, a high sensitivity platform for protein measurement, is a supplement to LC-MS. It can be equipped with automation systems to achieve high throughput which is important in late clinical stages. LBA is a reagent binding-based assay, and its performance highly depends on the quality of binding reagents, i.e., capture and detection reagents. Generating binding reagents with good affinity and specificity may be challenging and time-consuming. Different batches of binding reagents may be generated during the development time span, and great efforts are needed to ensure the consistency in reagent characteristics and hence performance of the LBA.

### Bioanalytical Evaluation of Shedding

Whereas biodistribution refers to the spread of viral gene therapeutics within the body system, shedding is the release of the virus-based gene therapeutics through secreta, excreta, or skin of the patient. Shedding studies assess the potential risk to the environment and the impact to untreated human and other species. The US FDA expects the applications of gene therapeutics (e.g., INDs, BLAs, and supplements to BLAs) to be accompanied by an environmental assessment unless a claim of categorical exclusion is granted under 21 CFR 25.15(a) (69).

Replication competency is an important factor in shedding evaluation. After its administration into the patient, the replication competent virus that can integrate with the genome of target cells amplifies within the patient’s body over time and increases the potential risk of shedding. To minimize shedding, most vectors are engineered to be conditional replication-competent or replication-incompetent. The investigation of any potential replication-competent recombinants should be conducted during the manufacture of replication-incompetent vector/gene therapeutics.

In the shedding study, the bioanalytical assay can be designed to detect either nucleic acids or infectious virus. A suitable bioassay should generate high-quality data that accurately represent the shedding profile of the GT and can be successfully applied to assess the risk of potential transmission to untreated individuals (70). At least one shedding bioassay should be quantitative. PCR and hybridization ELISA are common methods because of their high sensitivity, well-developed assay platform, fast turnaround, and high-throughput format. Both assays can be used to quantitatively determine the number of genome copies for shedding evaluation through the detection of the nucleic acids. The main disadvantage of nucleic acid-based assays is that they cannot differentiate the intact infectious virus and degraded non-infectious virus. Thus the detection of viral nucleic acids is not sufficient to infer the existence of infectious virus in shedding evaluation. Infectivity assay that titrates the viral material into the cells *in vitro* for a 50% infective dose can be used to measure the amount of infectious virus in the study samples. The advantage of infectivity assay is that it only detects the intact and infectious virus. Limited by the nature of the cell-based assays, the infectivity assay is inherently less sensitive than PCR and hybridization ELISA assays. A stepwise approach to analyzing shedding samples by combining nucleic acid-based and infectivity assays is recommended. Thus, PCR or hybridization ELISA method as a quantitative approach specific for nucleic acids of virus can be used as tier 1 of sample analysis. Study samples showing a high concentration of nucleic acids are further analyzed by infectivity assay in tier 2.

### Bioanalytical Evaluation of Immunogenicity

The immunogenicity of GTs requires the investigation of vector, transgene, and expressed transgene proteins because each component may contribute to shaping the host immune response (71). Pre-existing antibodies that can impact the postdose response, transgene delivery, and expression are evaluated prior to the administration of GTs (72). Developing a bridging ADA assay for total antibodies in serum or plasma can be challenging. The vector has a limited number of amino groups for the successful conjugation of biotin or sulfo-tag group. An undesirable labeling ratio can limit assay sensitivity. In addition, a positive control should be generated with high specificity to prevent a nonspecific signal to genetic materials of the host.

The transgene specific immunogenicity includes the immune response to transgene and transgene expressed protein. While the transgene is usually a lesser concern in immune response due to the small size and low immunogenicity of nucleic acids, the immunogenicity of the expressed protein should be closely monitored in humoral and cellular levels. For humoral immunogenicity assessment, LBA-based total antibody assay is the most popular method because of its high sensitivity and well-developed assay format. In comparison, flow cytometry and ELISPOT are often used to measure cellular immunogenicity. The cell-based NAb assay is recommended for analyzing the ADA positive samples. Additional characterization such as isotyping and cross-reactivity testing may be considered for immunogenicity risk assessment.

## CELL THERAPY

Cell therapy products have been on the US market for more than a decade (73), and chimeric antigen receptor T cells (CAR-T) are bringing new hope to cancer therapy, notably CD_19_ CAR-T for treating leukemia (Table IV). In short, blood is extracted from a patient (autologous) or a donor (allogeneic). T cells will be purified, expanded, and gene transfected using lentiviral or retroviral vectors *in vitro*. Lentiviral or retroviral vectors introduce a single chain variable fragment (ScFV) antibody which is specific to the antigen, e.g., CD19, into the cell surface of CAR-T. Upon administration to the patient, CAR-T will bind to tumor cell surface antigens through ScFV and kill the tumor cells. CAR-T cell kinetics is vital to evaluate the proliferation and persistence of infused cells. CAR-T cell number in blood, bone marrow, and other tissues is usually measured by flow cytometry. Since CAR-T is genetically modified, the copy number of exogenous gene should be assessed prior to and during the CAR-T cell administration: a certain transgene copy number in a given cell population is required for efficient treatment, but a higher copy number may lead to greater risk of genotoxicity. CAR-T transgene copy number assessments are usually conducted via qPCR. Exogenous expression of ScFV raises the concern of immunogenicity of CAR-T; hence, conventional bridging assay is applied for anti-ScFV antibody analysis. Non-cell-based neutralizing assay is used for the analysis of the neutralizing activity of ScFV based on its binding activity with the antigen.

**Table 4:** Some US FDA-Approved Cell and Gene Therapy Products

Approval year	Generic name	Trade name	Sponsor	Indication
2010	Sipuleucel-T	Provenge	Dendreon	Advanced prostate cancer
2015	Talimogene laherparepvec	Imlygic	Amgen	Metastatic melanoma
2017	Tisagenlecleucel	Kymriah	Novartis	Acute lymphoblastic leukemia
2017	Axicabtagene ciloleucel	Yescarta	Kite	Non-Hodgkin lymphoma
2017	Voretigene neparvovec-rzyl	Luxturna	Spark	Inherited retinal dystrophy
2019	Onasemnogene abeparvovec-xiol	Zolgensma	AveXis/Novartis	Spinal muscular atrophy in children
2019	Autologous cultured chondrocytes on porcine collagen membrane	MACI	Vericel	Symptomatic cartilage defects of the knee
2020	Brexucabtagene autoleucel	Tecartus	Kite/Gilead	Mantle cell lymphoma (MCL)

### CAR-T Cell Number Analysis Using Flow Cytometry for Pharmacokinetics

For the cell kinetics analysis by flow cytometry, an antibody recognizing the extracellular portion of the CAR, followed by a fluorescently conjugated secondary antibody, is employed to measure the CAR-expressing cells. The distribution and persistence of these CAR-expressing cells can also be analyzed in total T cells and subsets of T cells to understand their behavior and dynamics. Potential variables in the clinical samples including the shedding target, cell viability, blood cell concentration, etc. could affect the readout and should be carefully evaluated in the planning stage. The required format of readout, i.e., as percentage or absolute count, also should be considered.

In the absence of regulatory guidance for CAR-T PK bioanalysis, a fit-for-purpose approach is generally followed. Relative accuracy and precision are assessed by generating a series of “nominal samples” with a blank matrix spiked with CAR-T cells at different density. Specificity is confirmed when screening the critical reagents and validated using a negative control, either isotype control or fluorescent minus one (FMO). Sample storage stability should be assessed according to the clinical setting at room temperature or 2–8°C for the duration of shipment to the analytical laboratory. Mock samples generated by spiking CAR-T cells in whole blood or incurred clinical samples can be used for this assessment.

### CAR-T Gene Copy Number Analysis Using qPCR for Pharmacokinetics

For CAR-T bioanalysis via qPCR, the following points should be considered: 1) The dynamic range for CAR-T copy number determination must be defined. Ideally, the dynamic range should cover 5 to 6 orders of magnitude (74). A typical standard curve is prepared by spiking serial CAR-T plasmids at different concentrations into genomic DNA extracted from blood. 2) Sensitivity, defined by the lower limit of quantification (LLOQ), is usually assessed by examining serial dilutions (e.g., 10-fold) of CAR-T plasmids. A demonstrated limit of quantification ≤ 50 copies/μg genomic DNA is recommended by the FDA for qPCR assay applied to transgenes detection (75). 3) The Minimum Information for Publication of Quantitative Real-Time PCR Experiments (MIQE) guidance is a valuable reference for accuracy and precision assessment (74). 4) A negative control must be included for CAR-T measurement via qPCR assay. For this purpose, the genomic DNA without spiked CAR-T plasmid can be used as the template of PCR assay. 5) DNA purification may affect the accuracy of determined DNA VCNs. An internal gene control such as CDKN1A, albumin, or GAPDH has been used to calibrate genomic DNA concentration. In that situation, a secondary standard curve is often prepared (76, 77).

### Replication-Competent Lentivirus (RCL) Copy Number Analysis Using qPCR

During CAR-T manufacturing or subsequent administration, replication-competent lentivirus (RCL) can be generated by homologous or non-homologous recombination that may pose a health risk. Therefore, a CAR-T product must be tested for RCL prior to use in patients. Cell-based assays, while recommended by the FDA for RCL detection, may take up to 6 weeks for results. In this case, a qPCR assay may offer a quick alternative. Satisfactory bioanalytical parameters in RCL determination by qPCR have been reported (78) based on detection of envelope gene sequences (vesicular stomatitis virus G glycoprotein or VSV-G) for RCL in accordance with MIQE guidelines. The use of DMSO as an additive has been shown to increase assay sensitivity (78).

### Immunogenicity Considerations

Similar to other therapeutic proteins, the ADA detection method for CAR-T is based on the bivalent character of antibody binding. Briefly, ScFV were labeled with Biotin and Ruthenium which were added into the real samples or controls, respectively. Once there is ADA in the real sample or PC in the controls, the complex of “biotinylated-ScFV-ADA-ScFV-ruthenylated” will be formed. To ensure detection of all clinically relevant antibodies, it is recommended that screening and confirmatory ADA assays achieve a sensitivity of 100 ng/mL or better, which could be challenging due to the low affinity of the anti-ScFV.

The recombinant ScFV may structurally differ from the membrane-bound ScFV expressed on CAR-T cells. Consequently, immunogenicity to CARs may be missed due to the artificial nature of a ligand binding assay setup. T-cell lines expressing ScFV that offers the opportunity to measure anti-drug antibodies to the CAR in its natural cell environment have been developed (79) for use in flow cytometry as an alternative to ligand-binding assays. Humanized Anti-CAR19 antibodies in positive control samples (PCs) or in human serum samples were captured by Jurkat cells (an immortalized cell line of human T lymphocyte cells) transduced with CAR19 lentiviral vector to express murine CAR19 (CAR19 cells). After an incubation step and washing away any unbound antibodies, a R-Phycoerythrin (PE) labeled anti-IgG/MF(ab′)2 fragment was added to the cells in addition to a viability dye (efluor 780). After incubation, additional washing steps and a fixation step, the cells incubated with PCs or serum samples were analyzed on a flow cytometer.

A non-cell-based CAR-T NAb assay is usually applied based on the interference between target and ScFV of CAR-T. The anti-ScFV antibody with neutralizing capability will block the binding of the target to ruthenylated ScFV and cause a signal decrease. In order to develop a NAb assay with adequate sensitivity, it is critical to have a positive control antibody with high affinity and good neutralizing effect. Drug tolerance may not be a concern in CAR-T NAb assay as the concentration of free ScFV in human serum is relatively low. Cell-based NAb methods for CAR-T can also be developed with appropriate CAR-T cells and target cells.

### Cytokine Analysis for CAR-T

Cytokine release syndrome (CRS) resulting from rapid immune activation induced by CAR-Ts is a significant, treatment-related toxicity. Commercially available immunoassay kits of different platforms including ELISA, MSD, Luminex, Singulex, and Simoa are used to determine cytokine concentrations in variable sample types. Multiplexed cytokine panels can provide results for several analytes from the same sample simultaneously.

## THERAPEUTIC FUSION PROTEIN (TFP)

Therapeutic Fusion Proteins (TFPs) are engineered proteins linked to another molecule to form two (or more) component molecules which will maintain the biological function of the core domain. Compared to its parent protein drug, a TFP could provide better efficacy and/or safety characteristics through its partner domains, such as human IgG1 Fc protein, albumin, polyethylene glycol (PEG), or transferrin. The unique design of TFPs will help extend the circulating half-life of the drug, enhance pharmacology or novel mechanisms of action, and generate less immunogenic response compared to related antibodies (80–82). Since the introduction of etanercept in 1998, more than 20 TFPs have been approved by the FDA to meet heretofore unmet medical needs (Table V). TFPs are usually therapeutics with an endogenous counterpart, e.g., THF-alpha receptor and VEGF receptor. Among these TFPs, ten of them are Fc-fusion proteins which are attracting more attention from researchers on cytokine pathways.

**Table 5:** Some US FDA-Approved Therapeutic Fusion Proteins

Approval year	Generic name	Trade name	Key structure	Sponsor	Indication	Mechanism of action
1998	Etanercept	Enbrel	Tumor necrosis factor (TNF) receptor fused to IgG1 antibody	Amgen	Rheumatoid arthritis	Interferes with tumor necrosis factor by acting as a TNF inhibitor
1999	Denileukin diftitox	Ontak	Interleukin-2 fused to diphtheria toxin	Eisai	T-cell lymphoma whose malignant cells express CD25	Binds to interleukin-2 receptors and introduce diphtheria toxin into cells
2001	Darbepoetin alfa	Aranesp	Erythropoietin 2 new sites fused with N-linked carbohydrate addition	Amgen	Anemia associated with chronic renal failure	Binds and activates Epo receptor
2001	Pegylated interferon alfa-2b	PegIntron	IFN-a-2b fused to 12-kD monomethoxy PEG	Merck	Hepatitis C and melanoma	Binds to IFN-a receptor 1 and 2 to affect immuno response and cell apoptosis
2002	Pegylated interferon alfa-2a	Pegasys	IFN-a-2a fused to monomethoxy PEG	Roche	Hepatitis C and hepatitis B	Binds to IFN-a receptor 1 and 2 to affect immuno response and cell apoptosis
2002	Pegfilgrastim	Neulasta	Granulocyte colony-stimulating factor fused to PEG	Amgen	Febrile neutropenia	Stimulates the production of white blood cells
2003	Alefacept	Amevive	Lymphocyte function associated antigen-3 (LFA-3) fused to IgG dimer	Astellas	Moderate to severe chronic plaque psoriasis	Inhibits the binding of endogenous LFA3 to CD2 cells interfering with activation of memory T cells
2005	Abatacept	Orencia	Interleukin-2 fused to diphtheria toxin	Bristol Myers Squibb	Highly active and progressive rheumatoid arthritis (RA)	Binds to the costimulatory molecules CD80 and CD86 on antigen-presenting cells (APC)
2008	Rilonacept	Arcalyst	IL-1 receptor extracellular domains fused to the Fc portion of human IgG1	Regeneron	Familial cold-induced autoinflammatory syndrome (FCAS)	Binds to and neutralizes IL-1 before it can bind to cell surface receptors
2008	Romiplostim	Nplate	14 amino-acid peptides fused to the human IgG Fc domain	Amgen	Immune thrombocytopenia	Binds to and activates the thrombopoietin receptor
2011	Belatacept	Nulojix	ECD of cytotoxic T-lymphocyte-associated antigen 4 (CTLA-4) fused to human IgG Fc	Bristol Myers Squibb	Post-transplantation lymphoproliferative disorder positive for Epstein-Barr virus (EBV)	Inhibits T-cell activation through costimulation blockade
2012	Aflibercept	Eylea	ECD of VEGF receptors 1 and 2 fused to human IgG1 Fc	Regeneron	Wet macular degeneration	Binds to circulating VEGFs and acts like a “VEGF trap”
2014	Albiglutide	Tanzeum	Hormone (glucagon-like peptide-1 dimer albumin fusion) fused to albumin	GlaxoSmithKline	Type 2 diabetes	Acts as an agonist at the GLP-1 receptor which causes an increase of insulin secretion
2014	Peginterferon beta-1a	Plegridy	IFNb-1b fused to PEG	Biogen Idec	Multiple sclerosis	Interferon beta leads to a reduction of neuron inflammation
2014	Dulaglutide	Trulicity	GLP-1(7-37) fused to human IgG4 Fc	Eli Lilly	Type 2 diabetes	Binds to glucagon-like peptide 1 receptors and increases insulin secretion by pancreatic beta cells
2015	Asfotase-alfa	Strensiq	Tissue-nonspecific alkaline phosphatase (TNSALP) fused with hIgG1 Fc	Alexion	Enzyme replacement therapy for onset hypophosphatasia	Replaces TNSALP enzyme which is responsible for formation of an essential mineral in normal bone
2015	Etanercept-szzs	Erelzi	TNF receptor 2 fused to human IgG1 Fc	Novartis	Autoimmune diseases	Reduces the effect of naturally present TNF
2018	Tagraxofusp-erzs	Elzonris	Interleukin 3 (IL-3) fused to truncated diphtheria toxin	Stemline Therapeutics	Blastic plasmacytoid dendritic cell neoplasm (BPDCN)	Binds to IL-3 receptors and affects its pathway
2018	Moxetumomab pasudotox	Lumoxiti	Anti-CD22 antibody fused to PE38 toxin	AstraZeneca	Relapsed or refractory hairy cell leukemia (HCL)	Binds to CD-22
2019	Luspatercept	Reblozyl	Human activin receptor type IIb (ActRIIb) linked to IgG1	Celgene	Anemia in beta thalassemia	Binds TGF superfamily ligands to reduce SMAD signaling

*PEG* polyethylene glycol, *TNF* tumor necrosis factor, *TNSALP* tissue-nonspecific alkaline phosphatase, *SMAD* small mothers against decapentaplegic, *ECD* extracellular ligand-binding domain, *VEGF* vascular endothelial growth factor

### Bioanalytical Challenges and Considerations

The method for PK bioanalysis of TFPs should be developed based on the nature of the protein such as size and structure, availability of the critical reagents, interference from soluble proteins, and the required assay sensitivity. Both LBA and LC-MS/MS are commonly used. For the LBA method, the main challenge is the presence of endogenous analyte, specific binding proteins, and nonspecific matrix components in the circulation that can cause substantial interference. For example, the endogenous level of growth hormone binding protein (GHBP) could range from ~200 to 5000 pmol/L in the blood samples of patients with growth hormone deficiency (GHD) diseases which could impede the analysis of the drug pegylated human growth hormone (PEG-hGH). To alleviate this problem, Myler *et al.* (83) introduced an acid treatment to dissociate PEG-hGH from serum endogenous GHBP. A 10-fold molar excess of GHBP was found to decrease PEG-hGH detection by >90%, while acid dissociation was shown to recover >80% of the analyte. On the other hand, acid treatment is not needed when the concentration of the soluble proteins in plasma or serum is low and the resulting interference insignificant. For example, the endogenous level of IL-2 is <30 pg/mL in normal serum or plasma and will not interfere with the analysis of denileukin diftitox using LBA where the assay sensitivity (LLOQ) is >100 pg/mL (84).

Strategies to improve assay sensitivity for LBAs may include the use of buffers with different blockers such as sheep and mouse sera to reduce the interference by human-anti-mouse-antibodies (HAMAs) and incubation time from 2 to 3 h to overnight. Washing steps after incubation with capture reagents should be avoided in order to prevent signal reduction caused by the dissociation of the weakly formed complex of capture reagents with the analyte (85). In a LC-MS/MS method for ethanercept in human serum, Iwamoto *et al.* (86) used a nano-surface and molecular-orientation limited (nSMOL) proteolysis technology which has a unique two-solid-surface assisted Fab-selective proteolysis by trypsin immobilized on the surface of nanoparticles (200-nm diameter) for protein bioanalysis which is oriented by the binding antibody Fc via Protein A/G in a pore (100-nm diameter). This approach focuses on analyzing the core proteins of interest while avoiding the extra peptides generated by the drug or carryover from matrices.

### Immunogenicity Considerations

The assessment and mitigation of the immunogenicity of therapeutic proteins have been reviewed (82, 87–89), and it has been reported that TFPs, especially Fc fusion protein, tend to show a lower incidence of developing immunogenicity (82). For example, etanercept has less immunogenic potential compared to anti-TNF monoclonal antibodies (90). A confirmation assay is recommended to evaluate whether the ADAs target the whole molecule or bind to a clinically relevant domain of TFPs. For example, the ADA method for denileukin diftitox should have one for measuring reactivity directed against intact denileukin diftitox and another to measure whether the confirmed ADAs are against the IL-2 portion of the drug. Patients with rheumatoid arthritis may have a high incidence of rheumatoid factor (RF) in their blood that could bind to Fc protein from a TFP. This point should be considered when developing an ADA method to analyze samples from these patients. Adding human IgG and anti-human IgM to the assay buffer could reduce the interference from RF (91).

## SUMMARY AND FUTURE PERSPECTIVE

The emergence of novel therapeutic modalities in the past 20 years has changed the status quo of the bioanalytical arena leading to the development and maturation of a diverse suite of technologies. Depending on the structure of the molecule, endogenous counterpart interference, and matrix protein-protein interaction requirements, LC/MS, LC-HRMS, LBA, and flow cytometry remain as widely used bioanalytical tools. For oligonucleotides and gene therapy, new methods such as qPCR, branched DNA, hybridized immunoassay, and ELISPOT have been added to the toolbox. The complex structures and diverse mechanisms of action of new drug modalities have raised significant challenges to PK, PD, and immunogenicity evaluations such as the relevance of measuring the “total vs free” and “total vs intact” portion of the biotherapeutics. Immunogenicity assessment may require more explicit evaluation of the intact molecule, each function domain, linkers, the degraded form, metabolites, and the combination thereof. While the continued demand for greater sensitivity, selectivity, detectability, drug tolerance, speed, and cost efficiency is driving the development of new tools such as ultrasensitive and multiplex equipment, a balance needs to be achieved between the use of new and current approaches in the absence of regulatory guidance on the former in order to mitigate the risks carried forward on critical aspects such as data quality, data comparability, and data integrity (92, 93).
